# Acute Heart Failure in Sub-Saharan Africa: An Underrecognized Cause of Mortality

**DOI:** 10.7759/cureus.111626

**Published:** 2026-06-27

**Authors:** Sidi Mohamed Limame, Mohamed Issa Elkharchi

**Affiliations:** 1 Cardiology, Centre National de Cardiologie, Nouakchott, MRT

**Keywords:** acute heart failure, mauritania, morbidity, mortality, sub-saharan africa

## Abstract

Acute heart failure (HF) remains a major public health challenge in sub-Saharan Africa, contributing substantially to morbidity and mortality. Despite its burden, the epidemiological characteristics of acute HF in the region remain incompletely understood. The experience of the National Center of Cardiology in Mauritania demonstrates that optimal management of acute HF is achievable within a tertiary care setting; however, significant improvements are still needed across the rest of the country. Moreover, simple interventions focused on prevention, early diagnosis, and patient education have the potential to improve outcomes in this serious condition, which has long been neglected in sub-Saharan Africa. This editorial highlights the need to recognize acute HF as a public health emergency. Given its considerable socioeconomic impact, the establishment of a multinational fund by sub-Saharan African governments to support a coordinated regional HF strategy is also warranted.

## Editorial

The results of a hospital registry of acute heart failure (HF) conducted at a tertiary care center in Mauritania were recently published [1]. Acute HF is a major public health problem worldwide; however, its characteristics in Mauritania remain poorly understood. The objective of this study was to evaluate the characteristics of acute HF in Mauritania.

We conducted a prospective, descriptive, single-center observational study that included patients hospitalized with acute HF at the National Center of Cardiology in Mauritania between January 1 and May 31, 2024. A total of 307 patients were hospitalized for acute HF. The mean age was 59.8 ± 15 years, and 64.8% were men. Hypertension was the most common cardiovascular risk factor (31.6%). New York Heart Association (NYHA) functional classes III and IV accounted for 12.7% and 73.6% of cases, respectively. Atrial fibrillation was present in 23.1% of patients, and anemia in 51.8%. The mean left ventricular ejection fraction was 41.6%. Decompensated chronic heart failure was the most common presentation (37.8%). Heart failure with reduced ejection fraction accounted for 55.7% of cases, while ischemic heart disease was the underlying etiology in 33.2%. Guideline-directed quadruple therapy was initiated in most patients before hospital discharge. The in-hospital mortality rate was 5.5%.

Acute HF in Mauritania primarily affects middle-aged individuals. Management at the National Center of Cardiology is largely consistent with international guidelines; however, substantial efforts are required to expand this level of care nationwide, where HF management remains inadequate [1].

The epidemiology of HF in Africa has been assessed through several national registries. In Nigeria, Ogah et al. reported on a cohort of 452 patients hospitalized with acute HF and found that patients were younger than those in developed countries. HF was more common in men and was frequently associated with severe symptoms due to delayed presentation. Hospital mortality was comparable to that reported in other regions worldwide [2].

Similarly, the Tanzania Heart Failure (TaHeF) Registry included 427 patients and demonstrated that individuals with HF were younger than those in high-income countries. The etiologic profile of HF is evolving, with hypertension becoming increasingly prevalent and acute rheumatic fever declining in importance. Anemia, atrial fibrillation, and illiteracy were identified as modifiable predictors of mortality [3].

In Uganda, the Mbarara Heart Failure Registry (MAHFER) enrolled 215 patients. Overall mortality among HF patients was approximately twice that of the general population, while the use of evidence-based HF therapies remained suboptimal [4].

The Douala Heart Failure (Do-HF) Registry in Cameroon included 347 participants and found that nearly half of the patients died within 36 months of follow-up. Late diagnosis and poverty were associated with poor outcomes. The authors recommended prioritizing prevention strategies focused on earlier diagnosis and socioeconomic determinants to improve HF prognosis [5].

The Kenyan National Heart Failure Registry included 795 patients. Participants were predominantly middle-aged and commonly presented with recent-onset symptoms and reduced left ventricular ejection fraction. The in-hospital mortality rate was 6.6% and was independently associated with age greater than 50 years and advanced HF severity. The authors emphasized the need for earlier screening and more intensive initial management of HF [6].

Multinational hospital registries have also provided important insights into HF across Africa. The Sub-Saharan Africa Survey of Heart Failure (THESUS-HF) I study enrolled 1,006 patients with acute HF. The study demonstrated that acute HF in sub-Saharan Africa is predominantly non-ischemic in origin, most commonly attributable to hypertension. It affects middle-aged adults of both sexes and is associated with substantial mortality [7].

The International Congestive Heart Failure (INTER-CHF) study included 5,813 patients with HF, of whom 1,294 were from Africa. The study highlighted considerable regional variation in socioeconomic and clinical characteristics, etiologies, and treatment patterns among African patients with HF. It recommended improving health insurance coverage and access to essential medications [8].

The anticipated results of the THESUS-HF II study, which enrolled patients from approximately 50 hospitals across 16 African countries, are expected to provide valuable information on the epidemiology of acute HF in Africa and help identify healthcare priorities to improve its management [9].

In sub-Saharan Africa, HF accounts for 9.4% to 42.5% of hospital admissions [10]. Although precise data on the number of tertiary healthcare facilities are unavailable, more than 4,900 public hospitals have been identified across the region [11]. The Pan-African Society of Cardiology's strategy for combating HF includes training multidisciplinary teams in HF management [12], implementing standardized diagnostic algorithms, and strengthening education programs adapted to the realities and resource limitations of sub-Saharan Africa [13].

In conclusion, HF in sub-Saharan Africa should be regarded as a public health emergency. Its substantial socioeconomic burden justifies establishing a multinational fund to support a coordinated regional HF strategy across sub-Saharan African countries. The Mauritanian acute HF registry demonstrates that optimal care can be delivered at a tertiary referral center in the capital city; however, significant improvements are still needed throughout the rest of the country. Simple interventions focused on prevention, early diagnosis, and education have the potential to improve outcomes in this serious condition, which has long been neglected in sub-Saharan Africa.
